# Effectiveness of nurse-led interventions on psychological well-being among older adults: A systematic review and meta-analysis

**DOI:** 10.6026/973206300212319

**Published:** 2025-08-31

**Authors:** Jayalakshmi Lakshmanan, Bhuvaneswari Gopalakrishnan, Shankar Shanmugam Rajendran, Kumaresan Cithambaram, Munikumar Ramasamy Venkatasalu, Mala G., Rajeswari Subramanian, Nithin George, Nirmala Asaithambi, Periyanayagi Palavesam

**Affiliations:** 1 Department of Mental Health Nursing, Saveetha College of Nursing, Saveetha Institute of Medical and Technical Sciences, Chennai, Tamil Nadu, India; 2 Department of Community Health Nursing, Saveetha College of Nursing, Saveetha Institute of Medical and Technical Sciences, Chennai, Tamil Nadu, India; 3 Department of Paediatric Nursing, College of Nursing, Madras Medical College, Chennai, Tamil Nadu, India; 4 Department of Nursing and Healthcare, Technological University of the Shannon, Athlone, Ireland, Europe; 5 Faculty of Health Sciences, University of Greenwich - University of Nottingham, Nottingham, England; 6 School of Health, Medicine and Life Sciences, University of Hertfordshire, Hertfordshire, AL10 9AB, United Kingdom; 7 Department of Pediatric Nursing Saveetha College of Nursing, Saveetha Institute of Medical and Technical Sciences, Chennai, Tamil Nadu, India; 8 School of Health, Medicine and Life Sciences, University of Hertfordshire, Hertfordshire, AL10 9AB, United Kingdom; 9 Meenakshi Academy of Higher Education and Research, No: 12, Vembuliamman Koil Street West K.K. Nagar, Chennai, India; 10 Department of Community Health Nursing, Omandurar Medical College Hospital, Chennai, India

**Keywords:** Older people, elderly, psychological well-being, systematic review, meta-analysis

## Abstract

Advancements in medical care have led to increased life expectancy, resulting in a growing older adult population facing challenges to their well-being. This study aims to systematically review the effectiveness of nurse-led interventions on the psychological well-being of older adults. A comprehensive review of studies from 2010 to 2024 found that these interventions significantly reduced depression, stress and anxiety, while improving self-esteem. The findings suggest that integrating nurse-led interventions into routine care could be beneficial for older adults. Future research should focus on standardizing these interventions and assessing their long-term impact.

## Background:

Over recent decades, advancements in medicine, public health, and socioeconomic development have substantially increased the life expectancy of older adults, with one billion people worldwide aged 60 and above in 2019 [1]. While this demographic shift offers various benefits, it also brings challenges such as physical and psychological decline, increased risks of social and economic exclusion, and a rise in mental health issues , the global population of individuals aged 60 years and older is expected to nearly double, with 80% residing in low- and middle-income countries [2, 3]. In India, the elderly population has grown from 20 million in 1951 to 83.58 million in 2006 and is projected to reach 173 million by 2026 [4, 5]. This rapid growth places older adults, especially those in developing countries, at increased risk for psychological issues [6]. The World Health Organization (2017) reported that one in six older adults' experiences abuse, which can lead to depression, anxiety and other long-term psychological consequences. Furthermore, mental health disorders account for 10.6% of disability-adjusted life years (DALYs) in this age group, with 27.2% of suicides occurring among older adults [7, 8]. Depression is particularly prevalent in India, with an estimated 34.4% of older adults affected, compared to the global prevalence range of 4.7% to 16% [9]. Elderly individuals in care homes face varying stress levels, with 30% reporting high stress and 46.7% moderate stress. Males, especially widowers, experience higher levels of anxiety and lower self-esteem, which negatively impacts their mental health [10]. These psychological issues not only affect the elderly directly but also place significant strain on healthcare systems and families [11]. In India, urbanization and economic changes have led to the rise of nuclear families, resulting in older adults living independently and relying more on healthcare professionals [12]. Mental health problems in this population necessitate comprehensive assessment and intervention, with nurses playing a crucial role in delivering person-centered care [13]. Studies have shown that nurse-led interventions can effectively improve both the physical and psychological health of older adults [13, 14-15]. However, there is a lack of synthesized evidence regarding the effectiveness of such interventions in enhancing psychological outcomes in this group. Therefore, it is of interest to assess the impact of nurse-led interventions on the psychological well-being of older adults, to provide clearer insights into their potential benefits.

## Methodology:

This systematic review included RCTs and quasi-experimental studies and the protocol for this systematic review was registered with the International Prospective Register of Systematic Reviews (PROSPERO) (Registration is withheld to protect anonymity). This review is reported in line with the Preferred Reporting Items for Systematic Reviews and Meta-Analyses (PRISMA).

## Search strategy:

The search strategy used the PICO (Population, Intervention, Comparison and Outcome) framework. In consultation with a subject librarian, search terms, including MeSH terms, were developed (Table 1 - see PDF). A comprehensive search for eligible studies was conducted across several databases, including the Cumulative Index to Nursing and Allied Health Literature (CINAHL), MEDLINE, PsycINFO and Web of Science, covering the period between 2010 and 2025. Additionally, manual searches were performed on reference lists and grey literature, such as Clinical Trials.gov, to ensure a thorough search. A language filter was applied to include only publications in English and the Boolean operators 'OR' or 'AND' were appropriately used to expand and narrow the search. In addition, truncations and wildcards were used depending on the database. The full search terms are listed in (Table 1 - see PDF).

## Eligibility criteria:

The inclusion and exclusion criteria were based on the PICO framework, with eligible studies including randomised and quasi-experimental studies involving participants aged 60 years or older who had received nurse-led interventions targeting psychological well-being, such as depression, anxiety, stress and self-esteem. Studies were excluded if full-text articles were unavailable, if non-nursing professionals conducted interventions, or if the articles were published in languages other than English.

## Study selection and screening:

Two independent reviewers (JL and RK) searched the database and the relevant studies were exported to Rayyan, screening software Duplicates were removed and two reviewers (JL and RK) performed the initial and full-text screening against the inclusion and exclusion criteria. A third reviewer (KK) was consulted if there were any disagreements and a consensus was achieved through open and transparent discussions.

## Data extraction:

One reviewer (JL) extracted the data and the second reviewer (KR) verified its accuracy. The following information was extracted using a standardized extraction form: authors, publication year, country, research setting, patients (sample size, mean age and sex), control groups, follow-up and outcome measures. Interventions (activity, facilitator, format, duration, length), frequency, adherence, adverse events, main outcomes and results (means and standard deviations). The authors of the selected articles were contacted via email to acquire any missing information; however, none of the authors responded to the email. Disagreements were resolved through discussion and a third reviewer was involved to (KC) reach a consensus.

## Quality appraisal:

The Cochrane risk-of-bias tool was used to assess the quality of the included RCTs and ROBINS-I for NRCTs. This encompasses biases related to selection and allocation, intervention/exposure administration, outcome assessment and detection and participant retention. Confounding, selection, intervention classification, deviation from intended interventions, missing data, outcome measurement and reporting biases were evaluated using the ROBINS-I tool for non-randomized controlled trials. Two reviewers (JL and RS) independently conducted the assessments and held meetings to ensure accuracy and consistency. A third reviewer (MRV) was involved in resolving disagreements and uncertainties and a consensus was reached through discussion.

## Analysis:

Meta-analysis was performed using the RStudio software. The mean difference (MDs), standard mean difference (SMDs) and the associated 95% confidence interval (CIs) were used to estimate the pooled effect size for each parameter. P < 0.05 was considered statistically significant and heterogeneity was assessed using the I2 statistic. When I2 was > 50%, the finding was regarded as heterogeneous and a random-effects model was used.

## Results:

The initial search generated 2,849 results, which were uploaded to the Rayyan screening software. After removing 468 duplicates, 2,381 papers remained. Of these, 2,351 were excluded based on the inclusion and exclusion criteria during the initial screening. Following this, 30 studies were identified for full-text review, and 24 studies were excluded for various reasons, including wrong study design (N=5), wrong outcomes (N=3), wrong settings (N=2), wrong study population (N=2), inconsistent scales (N=5), protocol (N=1), and intervention carried out by non-nursing professionals (N=6). Ultimately, six studies were included in the meta-analysis. Additionally, 21 reports were retrieved from other sources, including 19 from hand searches and one from a citation search. Two reports were included from these, while 18 studies were excluded due to wrong study design (N=5), inconsistent scales (N=4), wrong settings (N=2), absence of pre-test values (N=1), or non-nursing professionals carrying out interventions (N=6). In total, eight studies were included in the review. Figure 1 (see PDF) summarizes the search and screening process (PRISMA). The included studies were conducted in various countries, starting with Taiwan (Wu *et al.* 2011) [29] and followed by India (Metilda & Nalini, 2020) [4], Iran (Heidari *et al.* 2020) [25], Korea (Song & Boo, 2022) [27], and then more studies from India (Sahu, Mohanty, & Pahantasingh, 2019) [26], Taiwan (Yu *et al.* 2022) [28], and finally India again (Sheeja & Annie Chandra, 2024) [30] and Neha *et al.* 2018) [22] (Table 2 - see PDF). Participants were aged 60 years or older, with sample sizes ranging from 25 to 100 individuals. The interventions in these studies included laughter therapy, progressive muscle relaxation, guided imagery, reminiscence therapy, music performance, and tailor-made interventions. The quality of studies was assessed using the Cochrane risk of bias tool for randomized controlled trials (RCTs) and the ROBINS-I tool for non-randomized controlled trials (NRCTs). Most studies demonstrated a low risk of bias across multiple domains, including random sequence generation, allocation concealment, and blinding of participants and outcome assessors. However, some studies had unclear or missing data on incomplete outcome reporting (Table 3 - see PDF).

## Outcome Summary (Table 4 - see PDF):

Six studies measured depression, two measured stress, two measured anxiety, and two measured self-esteem. Statistically significant results were found for stress and anxiety in studies by Sheeja & Chandra (2024) [30] and Neha *et al.* (2018) [22], and for depression in studies by Heidari *et al.* (2020) [25], Metilda & Nalini (2020) [4], and others.

## Effect Sizes (Tables 5 - see PDF and 6 - see PDF):

Six studies assessed the impact of interventions on depression. The pooled standardized mean difference (SMD) for these studies was -6.24 (95% CI: -8.44, -4.05), indicating a significant reduction in depression scores. Heidari *et al.* (2020) [25] reported the largest effect size of -6.93 (95% CI: -8.05 to -5.82), while Sahu, Mohanty, and Pahantasingh (2019) [26] found the greatest effect size of -10.54 (95% CI: -12.75 to -8.32). There was significant heterogeneity (I^2^ = 94.5%, p < 0.0001), suggesting variability in the effects across studies. Wu *et al.* (2011) [29] and Sahu, Mohanty, and Pahantasingh (2019) [26] demonstrated significant improvements in self-esteem, with effect sizes of 6.99 (95% CI: 5.74 to 8.3) and 25.50 (95% CI: 20.28 to 30.73), respectively. However, the studies showed high heterogeneity (I^2^ = 97.8%, p < 0.0001). Sheeja & Annie Chandra (2024) [30] and Prahash & Sangeetha (2019) found significant reductions in stress, with pooled SMDs of 7.63 (95% CI: 6.58 to 8.67) and 4.57 (95% CI: 3.77 to 5.37), respectively. Significant heterogeneity (I^2^ = 95.2%, p < 0.0001) was observed across the studies. Sheeja & Chandra (2024) [30] reported a large effect of nurse-led interventions on anxiety reduction with an SMD of 7.24 (95% CI: 6.24 to 8.23). Neha *et al.* (2018) [22] also reported a significant effect on anxiety, with an SMD of 3.88 (95% CI: 3.17 to 4.59). The overall pooled effect was 5.54 (95% CI: 2.25 to 8.83) with high heterogeneity (I^2^ = 96.5%, p < 0.0001). These results suggest that nurse-led interventions have a significant impact on reducing depression, self-esteem issues, stress, and anxiety, though the magnitude of effects varies across studies and interventions.

## Discussion:

The systematic review and meta-analysis showed that nurse-led interventions are effective in improving psychological well-being in the elderly population by significantly reducing depression, stress and anxiety as compared to the control group and increasing self-esteem. This is in line with, Dixit and Lalitha (2022) study [17], which showed a significant decrease in the severity of depression symptoms at post-test. Equally, Klainin-Yobas *et al.* (2015) [18] demonstrated the beneficial effect of the structured reminiscence psychotherapy in decreasing the levels of depression, which was found to be statistically significant mean value of pre-test mean of 28.033 (SD = -6.593) and a post-test mean of 21.366 (SD = -6.436). Javadzade *et al.* (2024) [16] also highlighted the effectiveness of Mindfulness-Based Stress Reduction (MBSR) on decreasing depression and improving emotion regulation and sleep quality in elderly people suffering from depression (p<0.001). In addition to these findings, new evidence supports nurse-led interventions. The findings revealed a decrease in older adults' stress by nurse-led interventions and it was consistent with existing evidence. For example, Dixit and Lalitha (2022) [17] conducted guided imagery interventions in old age homes and found a large reduction in stress with pre-test scores of M = 57.475 (SD = 8.430) and post-test scores of M = 11 (SD = 4), which was a mean difference of 46.475. Similarly, Yobas *et al.* (2015) [18] showed that nurse-administered interventions were successful in reducing stress in elderly subjects. Additionally, Zheng *et al.* (2023) [23] found that a nurse-led positive psychological intervention (PPI) can effectively decrease stress and depression in older adult community-dwellers with MCI, but the intervention effects decreased during the follow-up period. Together, these studies demonstrate the beneficial and adaptable nature of nurse-led interventions regarding stress among elderly individuals and encourage the inclusion of these interventions in older adult care, improving their psychological well-being [21, 24]. The findings revealed that nurse-led interventions are effective in reducing anxiety among the elderly population. The findings are consistent with other studies, including Mehta and Siva (2019) [19], who found Jacobson's Progressive Muscle Relaxation (JPMR) technique significantly reduces anxiety in the elderly population. Maheshwari *et al.* (2021)'s [20] study also confirmed these findings and indicated that psycho-educational training significantly improved self-esteem (t= -19.64, p<0.001) and decreased depression, anxiety and stress in elderly people. However, despite its strengths, this study has several limitations. First, this review included studies published between 2010 and 2024, which may present limitations, as potentially relevant studies conducted outside this time frame or published in languages other than English were excluded. Second, the effects of the interventions on psychological outcomes in some cases were derived from very few studies, potentially limiting the generalizability of the findings. The heterogeneity of the studies was high, particularly concerning the type and duration of the intervention, which makes it difficult to generalize the effects of nurse-led interventions. Finally, this review only included interventions conducted by nurses, possibly excluding multidisciplinary approaches that could produce different results. This review has numerous implications for clinical practice, research and policy. First, nurse-led interventions positively affect psychological well-being, suggesting that these interventions are feasible, promising non-pharmacological interventions and appropriate for older adults. In the clinical context, nurse-led interventions can be embedded in organized and evidence-based care plans, particularly when access to specialist mental health services is restricted. Thus, these are feasible and patient-centred interventions which can be extended to different healthcare settings.

## Conclusion:

Nurse-led interventions can improve the psychological well-being of the elderly. However, high heterogeneity and methodological limitations warrant caution in interpreting the results. Further research with larger sample sizes, standardized interventions and longer follow-up is needed to confirm these findings.
